# Structural Organization of Enzymes of the Phenylacetate Catabolic Hybrid Pathway

**DOI:** 10.3390/biology4020424

**Published:** 2015-06-12

**Authors:** Andrey M. Grishin, Miroslaw Cygler

**Affiliations:** Department of Biochemistry, University of Saskatchewan, Saskatoon, Saskatchewan S7N 5E5, Canada; E-Mail: andrey.grishin@usask.ca

**Keywords:** phenylacetate degradation, catabolic pathway, *paa* operon, three-dimensional structure

## Abstract

Aromatic compounds are the second most abundant class of molecules on the earth and frequent environmental pollutants. They are difficult to metabolize due to an inert chemical structure, and of all living organisms, only microbes have evolved biochemical pathways that can open an aromatic ring and catabolize thus formed organic molecules. In bacterial genomes, the phenylacetate (PA) utilization pathway is abundant and represents the central route for degradation of a variety of organic compounds, whose degradation reactions converge at this pathway. The PA pathway is a hybrid pathway and combines the dual features of aerobic metabolism, *i.e.*, usage of both oxygen to open the aromatic ring and of anaerobic metabolism—coenzyme A derivatization of PA. This allows the degradation process to be adapted to fluctuating oxygen conditions. In this review we focus on the structural and functional aspects of enzymes and their complexes involved in the PA degradation by the catabolic hybrid pathway. We discuss the ability of the central PaaABCE monooxygenase to reversibly oxygenate PA, the controlling mechanisms of epoxide concentration by the pathway enzymes, and the similarity of the PA utilization pathway to the benzoate utilization Box pathway and β-oxidation of fatty acids.

## 1. Introduction

Aromatic compounds are abundant in nature and diverse in their chemical structure. Together with carbohydrates they are one of the most abundant sources of organic carbon [1] in the world. Compounds derived from petroleum such as benzene, toluene, ethylbenzene and xylene (BTEX) and others such as polychlorinated biphenyls (PCBs), polycyclic aromatic hydrocarbons (PAHs) and pentachlorophenol are among common environmental pollutants that contain an aromatic ring [2]. Plants lack the pathway for degradation of aromatic compounds and in the animal kingdom, there exist limited capacities for their utilization [3].

The degradation/utilization pathways for aromatic compounds are found predominantly in microbes allowing them to use aromatic compounds as the sole source of carbon [3]. Due to the high resonance energy of the aromatic system, these compounds have limited reactivity and are quite resistant to degradation. Nevertheless, microbes have evolved enzymatic pathways to accomplish this task, both under aerobic [4] as well as anaerobic conditions [5]. To access a wide variety of aromatic compounds, microbes have developed a strategy of converting them through the so-called “peripheral pathways” to a few central intermediates, therefore reducing the need to develop numerous specialized pathways [4]. Two aerobic strategies have evolved for breaking down aromatic rings. The classical aerobic strategy employs two multicomponent monooxygenases or a dioxygenase to introduce two hydroxyl groups into the aromatic ring, converting it first into catechol and subsequently cleaving the bond adjacent to the hydroxyls in an oxygen-dependent reaction [6]. The second strategy, discovered more recently for phenylacetate and benzoate catabolism [7], proceeds through attachment of the aromatic compound to coenzyme A, followed by its oxidation and ring opening. The initial mechanism postulated that the aromatic character of the ring was destabilized by the addition of two hydroxyl groups in two successive oxygenase steps [8]. However, several years later the conversion of the aromatic ring was proven to occur through a non-aromatic epoxide intermediate, a reaction catalyzed by a multicomponent monooxygenase [9,10].

The breakdown of aromatic compounds also occurs under anaerobic conditions whereby two independent metabolic strategies have evolved, both requiring the formation of a benzoyl-CoA intermediate. The first one reduces benzoyl-CoA by a class I reductase in a reaction driven by ATP hydrolysis [11]. The second, more recently discovered strategy uses ATP-independent class II benzoyl-CoA reductases that are multisubunit enzymes containing several cofactors [12]. Several reasons were proposed as to why processing of phenylacetate and benzoate in the form of CoA esters may be beneficial. CoA-bound intermediates are membrane impermeable, which keeps the degradation products contained within the cell. The large attachment in the form of a CoA-moiety significantly improves the recognition of a relatively small hydrophobic molecule. Finally, facultative anaerobes can degrade CoA-derivatives through anaerobic pathways in conditions of oxygen shortage.

In this review we focus generally on the hybrid phenylacetate utilization pathway and in particular on the three-dimensional structures of the Paa enzymes and the spatial coordination of reactions within this pathway through the formation of multiprotein complexes.

## 2. Phenylacetate Hybrid Degradation Pathway

The best characterized of the aerobic aromatic compound degradation pathways utilizing CoA is the phenylacetate degradation pathway, also known as a hybrid pathway, as it contains elements of aerobic and anaerobic pathways. This hybrid pathway exists in ~1/6 of currently sequenced bacterial genomes [9]. A parallel benzoate-CoA degradation hybrid pathway has been identified in an additional 5% of the genomes [4]. In conjunction with the peripheral pathways, this hybrid pathway serves as the central route for degradation of a wide variety of aromatic compounds such as 2-phenylacetate, 2-phenylethylamine, tropic acid, and styrene [7]. Genes associated with phenylacetate catabolism in *Escherichia coli* are located within three transcription units, the main one being the *paa* operon. They encode twelve enzymes or enzymatic subunits: PaaA, PaaB, PaaC, PaaE, PaaF, PaaG, PaaH, PaaI, PaaJ, PaaK, PaaY and PaaZ, a transcription regulator PaaX and a protein with unknown function PaaD (Table 1).

**Table 1:** General information about the enzymes from the phenylacetate degradation pathway.

Protein	Function	Structure	Organism	Homologue	PDB ID	Organism	Family/fold
PaaK	Phenylacetate-CoA ligase	2Y4O (PaaK2) 2Y27 (PaaK1)	Burkholderia cenopacia	Benzoate-CoA ligase	2V7B	Burkholderia xenovorans	Adenylate-forming domain, class I
PaaI	Phenylacetyl-CoA Thioesterase	1PSU	*E. coli*	PaaI-like protein	4M20	Staphylococcus aureus	Thioesterase group of hotdog superfamily
		1J1Y	Thermus thermophilus	4-hydroxybenzoyl-CoA thioesterase	3R37	Arthrobacter sp.	
PaaA	Catalytic subunit of 1,2-phenylacetyl-CoA epoxidase	3PW1	*E. coli*	BoxB	3Q1G	Azoarcus evansii	Bacterial multicomponent monooxygenase
				MMOH	4GAM	Methylococcus capsulatus	
PaaB	Bridging subunit of 1,2-phenylacetyl-CoA epoxidase	3EGR	Ralstonia eutropha				PaaB-like proteins
PaaC	Structural subunit of 1,2-phenylacetyl-CoA epoxidase	3PW1	*E. coli*	MMOH	4GAM	Methylococcus capsulatus	Bacterial multicomponent monooxygenase
PaaE	Reductase subunit of 1,2-phenylacetyl-CoA epoxidase			Phtalate Dehydrogenase	2PIA	Burkholderia cepacia	Class IA Reductase
PaaD	unknown			Duf59	3LNO	Bacillus Anthracis	Domain of the unknown function 59
PaaG	1,2-epoxyphenylacetyl-CoA isomerase	4FZW	*E. coli*	BoxC	2W3P	Burkholderia xenovorans	Enoyl-CoA isomerase; Crotonase superfamily
		3HRX	Thermus thermophilus				
PaaZ	Oxepin-CoA hydrolase			BoxD	No structure	Azoarcus evansii	N-terminal: NAD(P)+-dependent aldehyde dehydrogenase C-terminal: (De)Hydratase group of hotdog superfamily
				Aldehyde dehydrogenase	2VRO	Burkholderia xenovorans	
PaaJ	3-oxoadipyl-CoA/3-oxo-5,6-dehydrosuberyl-CoA thiolase	1ULQ	Thermus thermophilus	Acetyl-CoA acetyltransferase	4N44	Clostridium acetobutylicum	3-ketoacyl-CoA thiolase (thiolase I)
				ThlA2	4E1L	Clostridium difficile	
PaaF	2.3-dehydroadipyl-CoA hydratase	4FZW	*E. coli*	BoxC	2W3P	Burkholderia xenovorans	Enoyl-CoA hydratase; Crotonase superfamily
PaaH	3-hydroxyadipyl-CoA dehydrogenase	3MOG	*E. coli*				3-hydroxyacyl-CoA dehydrogenase
PaaY	2-hydroxycyclohepta-1,4,6-triene-1-carboxyl-CoA thioesterase			GK2848	3VNP	Geobacillus kaustophilus	
PaaX	Transcriptional repressor			PaaX-like protein	3LO9	Jannaschia sp.	PaaX-like proteins containing helix-turn-helix motif

All chemical reactions within this pathway have been defined, intermediates identified and enzyme functions assigned [9,13,14] (Schema 1). The pathway can be divided into two parts, the upper and the lower part. The lower part has enzymes that are homologous to well-characterized enzymes of fatty acid degradation found in the β-oxidation pathway. In the upper part of the pathway (Schema 1) the first enzyme is the phenyl-CoA acetate ligase (PaaK) [15], which is followed by a multicomponent monooxygenase complex consisting of four proteins (PaaABCE) [9,14,16]. PaaABCE converts PA-CoA (compound II) to the 1,2-epoxide derivative (compound III). This epoxide derivative is converted by the PaaG isomerase to an oxepin, an oxygen-containing heterocycle consisting of a seven-membered ring with three double bonds (compound IV), cleaved to 3-oxo-5,6-dehydrosuberyl-CoA semialdehyde (compound V) and oxidized by PaaZ to yield a β-keto C_8_-intermediate (compound VI).

**Schema 1:**
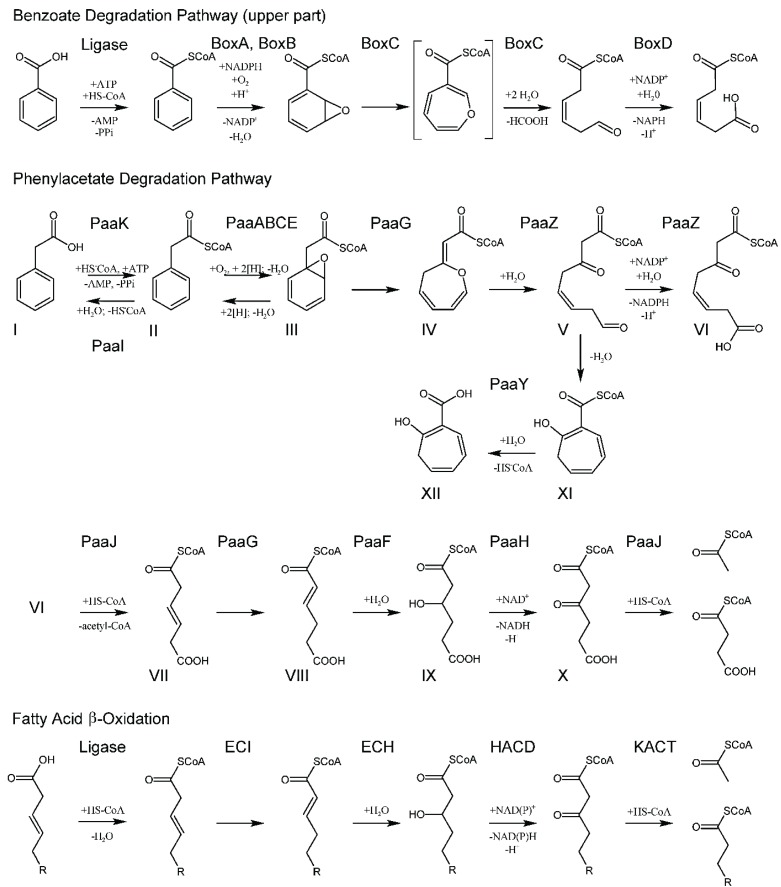
Paa phenylacetate degradation pathway. Comparison with Box pathway (**top**) and a fatty acid β-oxidation pathway (**bottom**). Compounds: I, phenylacetate; II, phenylacetyl-CoA; III, ring 1,2-epoxyphenylacetyl-CoA; IV, 2-oxepin-2(3H)-ylideneacetyl-CoA (oxepin-CoA); V, 3-oxo-5,6-dehydrosuberyl-CoA semialdehyde, VI, 3-oxo-5,6-dehydrosuberyl-CoA; VII, 3,4-dehydroadipyl-CoA; VIII, 2,3-dehydroadipyl-CoA; IX, 3-hydroxyadipyl-CoA; X, 3-oxoadipyl-CoA. Box pathway (top)—Compounds: (*from left to right*) benzoate; benzoyl-CoA; 2,3-epoxybenzoyl-CoA; the proposed oxepin-CoA is depicted in square brackets; 3,4-dehydroadipyl-CoA semialdehyde; 3,4-dehydroadipyl-CoA. Fatty acid β-oxidation pathway—Enzymes: ECI—Δ^3^-Δ^2^-enoyl-CoA isomerase; ECH—2-enoyl-CoA hydratase; HACD—3-hydroxyacyl-CoA dehydrogenase; KACT—3-ketoacyl-CoA thiolase.

In the lower part of the phenylacetate hybrid pathway, the C_8_-intermediate is cleaved by a thiolase (PaaJ), forming C_6_-dehydroadipyl-CoA (compound VII), which is isomerized to the α,β-unsaturated thioester 2,3-dehydroadipyl-CoA (compound VIII) by PaaG, hydrated by PaaF to 3-hydroxyadipyl-CoA (compound IX), and oxidized by NAD^+^-dependent dehydrogenase PaaH to 3-oxoadipyl-CoA (compound X) [9]. This final intermediate is cleaved by PaaJ to yield common metabolites, acetyl-CoA and succinyl-CoA [17].

## 3. The Upper Part of the Pathway

### 3.1. Activation of the Aromatic Compound

**Figure 1:**
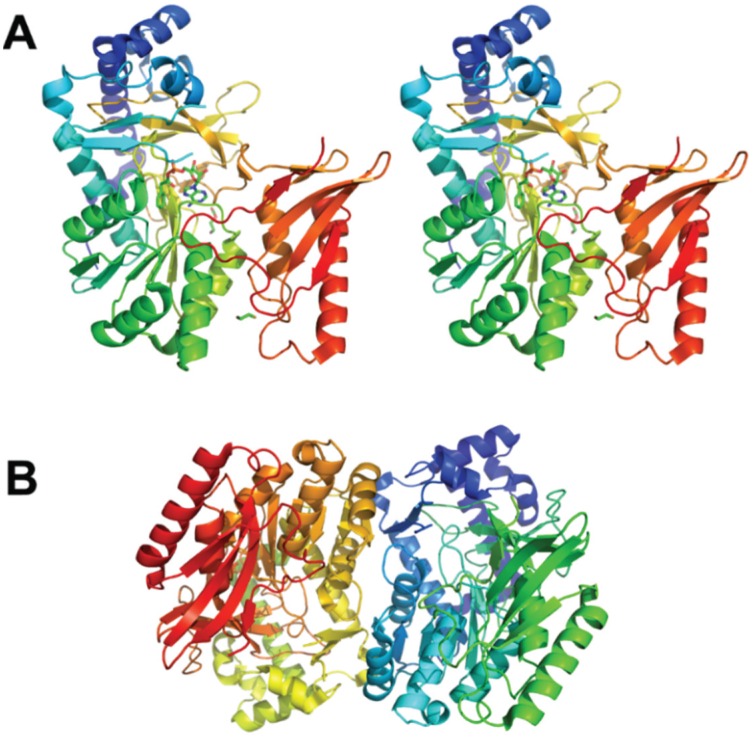
The crystal structure of PaaK1 from *Burkholderia cenocepacia*. (**A**) The stereoview of the PaaK1 monomer with the phenylacetyl adenylate intermediate painted in rainbow colors from blue at the N-terminus to red at the C-terminus. (**B**) Biological unit of PaaK is a dimer. This and other figures were prepared using PyMol (www.pymol.org).

PaaK catalyzes ATP and Mg^2+^-dependent attachment of phenylacetate to CoA, with AMP and PPi as other reaction products. PaaK belongs to the adenylate-forming enzyme superfamily, which includes enzymes activating short-to-long fatty acids, aromatic compounds, biosynthesis of peptide antibiotics and siderophores [18]. PaaK was shown to be relatively heat-stable and very specific toward phenylacetate [15,19]. The structure of PaaK (Figure 1) was determined for the enzymes from *Burkholderia cenocepacia*, which unusually possesses two PaaK enzymes, PaaK1 and PaaK2, but only a single copy of all other enzymes of the phenylacetate pathway. PaaK1 and PaaK2 share 69% identity over the entire length of enzymes, while the corresponding genes are located among other genes of the hybrid pathway. PaaK1 has lower K_m_ value for phenylacetate and a more relaxed substrate specificity than PaaK2, which likely arises from its extended aryl substrate pocket [20]. Nevertheless, the knockout of the *paaK1* gene alone does not impair growth and pathogenicity of the microbe [21].

### 3.2. Epoxidation of the Aromatic Ring

Monooxygenation of the aromatic ring is the most crucial step of the pathway. Gene-knockout experiments have shown that when any of the five genes *paaA*,*B*,*C*,*D* or *E* is deleted, the pathway is inactivated at this step [22]. However, experiments with co-expression of recombinant proteins from *Escherichia coli* [16] and *Pseudomonas* sp. strain Y2 [9], as well as pull-down experiments showed that only PaaA, B, C and E components are necessary for the oxidation reaction and formation of the epoxide. Not only could the full complex PaaABCE be purified [9] but PaaAC, PaaBC and PaaABC could also form stable subcomplexes [16]. The binding of the reductase subunit PaaE, despite being essential for the overall reaction, is the weakest in the overall complex. Subsequent structural and biochemical studies revealed that the monooxygenase contains a catalytic subunit PaaA, a structural subunit PaaC, a bridging subunit PaaB and a reductase paaE with the overall complex composition of PaaA_2_B_3-4_C_2_E_1_ as judged by SDS-PAGE. The complex contained six iron atoms of which two belonged to the iron-sulfur cluster of PaaE and four to the two molecules of PaaA [14].

**Figure 2:**
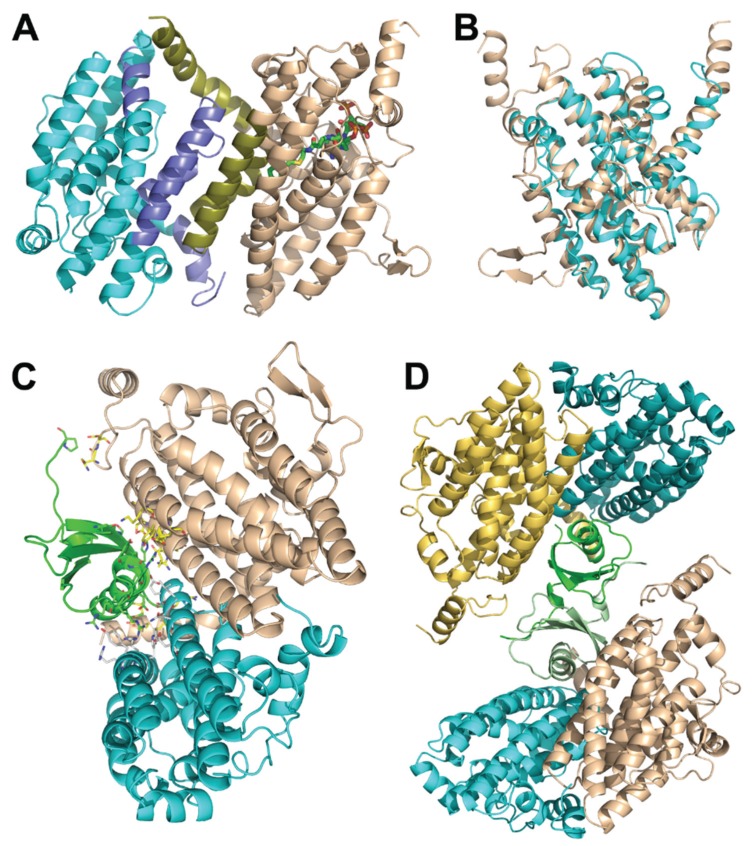
Paa monooxygenase complex. (**A**) The PaaA-PaaC heterodimer with bound phenylacetyl-CoA. The catalytic PaaA subunit is painted in a wheat color, the structural PaaC subunit is painted in cyan. (**B**) Superposition of PaaA (wheat) and PaaC (cyan); (**C**) The PaaACB heterotrimer. PaaA is painted wheat, PaaC is cyan and PaaB is green. The residues at the interface of PaaB with PaaA and PaaC are shown in stick mode. (**D**) The (PaaACB)_2_ heterohexamer. PaaA is painted wheat and olive, PaaC is cyan and light blue, and PaaB is green and light green.

The crystals of PaaAC were obtained in complex with several CoA derivatives, including the phenylacetyl-CoA substrate and the structures were determined at resolutions in the range 2.03–2.25 Å [23]. The PaaAC complex forms a heterotetramer (dimer of heterodimers) (Figure 2A). Although the PaaA and PaaC subunits display only 17% sequence identity, their structures share the same fold and can be superimposed with the root-mean-squares deviation of 1.7 Å [16] (Figure 2B). The PaaA-PaaC heterodimer interface has a significantly hydrophobic character, particularly for PaaA, explaining the insolubility of PaaA alone. Additionally, hydrophilic/charged interactions at the edges of the interface add to the stability of the complex. The association of the two heterodimers into a tetramer occurs solely through the interactions of the PaaC molecules from each heterodimer and is less extensive than the PaaA-PaaC interface [16].

The catalytic subunit in this complex is PaaA, while PaaC is thought to play a structural role. No iron ions were present in the PaaA structure; however, the expected iron binding residues Glu 42, Glu 72, His 75, Asp 126, Glu 155 and His 158 are very similar to those found in other BMMs [24]. Indeed, soaking the crystals with iron, although it significantly increased crystal disorder and decreased resolution, showed that the ions were bound in the expected site [16]. The phenylacetate-CoA binds to the PaaA subunit in a hairpin conformation within a ~20Å deep pocket in the protein. The adenine and phenyl moieties at the two ends of the substrate point in the same direction and the phosphates are at the hairpin bend near the protein surface (Figure 2A). The entrance to the substrate-binding pocket is partially covered by surface segments containing Lys103 and Leu286, which likely provide a dynamic door for substrate entry. The interaction of the substrate with Phe108 helps to orient the phenylacetyl moiety so that the aromatic C1–C2 bond becomes located exactly above the putative di-iron center and in the proximity to the unusual constellation of Lys68 coordinating three acidic residues, Glu49, Glu72 and Asp126, named the lysine bridge [16].

The information about the structure of PaaACB complex came from the investigation of these proteins in *Klebsiella pneumonia*. Although the crystal diffracted only to 4.3 Å resolution, the structure could be determined using the known high resolution models for all three components [25]. The stoichiometry of the three subunits in the complex is 2:2:2. The PaaAC subcomplex is the same as observed previously on its own while also containing the phenylacetate-CoA molecule bound to the PaaA subunit. The PaaB subunit binds to both PaaA and PaaC, subunits of one heterodimer in a cleft formed near to the PaaA/PaaC interface (Figure 2C). The heterohexamer is assembled through dimerization of two PaaB subunits (Figure 2D). This arrangement into a hexamer with the PaaB dimer in the middle and two PaaAC heterodimers on the outside was confirmed by negative stain electron microscopy and small angle X-ray scattering in solution. The structure of the heterohexameric PaaACB complex with two PaaAC lobes connected by the PaaB dimer displays a concave surface close to the phenylacetate-CoA binding site. The iron-binding α-helices C, E and F of PaaA are exposed on that surface. There is sufficient space for the molecule of PaaE reductase to bind to this surface near the substrate and participate in electron transfer. The lysine bridge, identified in the PaaA subunit near the di-iron center, was suggested as being involved in this transfer [25].

PaaE was characterized kinetically and spectroscopically [14,16]. It belongs to the class IA reductases [26], which are usually associated with dioxygenases, while classical monooxygenases, e.g., methane monooxygenase, utilize class IB or class III reductases. Indeed, advanced BLAST analysis identified the phthalate dioxygenase reductase from *Burkholderia cepacia* (PDB code 2PIA, [26]) as a structural homolog albeit with only a 22% sequence identity. PaaE contains an N-terminal NADPH- and FAD-binding domain and a C-terminal [2Fe-2S] ferredoxin-like domain and is thought to transfer electrons from NADPH through FAD and the iron-sulfur cluster to iron atoms in the active center of PaaA.

### 3.3. Ring Opening

In the next step of the degradation pathway, PaaG isomerizes 1,2-epoxyphenylacetyl-CoA epoxide (compound III), formed by the action of monooxygenase, to 2-oxepin-2(3H)-ylideneacetyl-CoA (compound IV) [9]. PaaG belongs to the crotonase fold superfamily, which also contains enoyl-CoA isomerases and enoyl-CoA hydratases involved in fatty acid β-oxidation. The structure of PaaG will be discussed below.

The next two steps, the hydrolysis of the oxepin and the oxidation of the terminal aldehyde group, are performed by the bifunctional enzyme PaaZ. It comprises two domains, the C-terminal R-specific hotdog fold hydratase (where R- and S- refer to the stereochemistry of the hydroxyacyl-CoA reaction product) and the N-terminal aldehyde dehydrogenase [13]. Despite different folds both R-specific hydratases, *i.e.*, PaaZ, and S-specific hydratases, such as PaaF (see below), utilize an acid-base catalytic mechanism.

In many microorganisms the *paaZ* gene contains only an aldehyde dehydrogenase domain instead of two above-mentioned domains, raising questions about the enzyme responsible for the ring opening. Such an enzyme, which performs oxepin-CoA hydrolysis (ring-opening), has been identified in *Aromatoleum aromaticum* as a hotdog fold hydratase coded by a gene outside the *paa* gene cluster [13]. This enzyme was also capable of hydrating crotonyl-CoA with high activity, suggesting that the hydrolysis of the oxepin-CoA may be a side reaction [13].

### 3.4. Controlling the Fate of Toxic Epoxide

Epoxides are active towards DNA and proteins [27] and significantly inhibit cell growth [28]. It is possible that some pathogenic bacteria, such as *Burkholderia cepacia*, have evolved to use epoxide against their hosts to promote pathogen survival and replication [29]. The fate of the toxic epoxide is controlled by several means. The most prominent safeguard is the ability of the PaaABCE complex to catalyze the reverse reaction—deoxygenation (distinguish from dioxygenation), in which 2H^+^ equivalents are used to yield phenylacetyl-CoA and water [14]. This PaaABCE-catalyzed reverse deoxygenation reaction occurs when the epoxide accumulates due to inadequate processing by the downstream enzymes PaaG and PaaZ.

The second control mechanism involves the thioesterase PaaI. It belongs to the hotdog fold superfamily [30] and catalyzes the breakdown of the early-stage metabolites of the Paa pathway (PA-CoA), reversing the action of PaaK. PaaI has a very narrow substrate specificity limited to PA-CoA [14], suggesting that PaaI removes the excess of PA-CoA and prevents overloading the capacity of PaaABCE.

Exploring the reaction catalyzed by PaaZ, Teufel and colleagues have found that oxepin-CoA spontaneously rearranges to 2-hydroxycyclohepta-1,4,6-triene-1-formyl-CoA (compound XI), which inhibits ring cleavage by paaZ [13]. However, the inhibitory activity of the compound XI is mitigated by PaaY, which was found to be able to hydrolyze this product to yield compound XII with the highest specificity among all reagents tested to date. A homolog of PaaY from *Geobacillus kaustophilus* HTA426 with 33% sequence identity has been structurally characterized (PDB code 3VNP) providing information about the overall structure of PaaY. Further studies revealed that PaaY associates into trimers, contains Ca^2+^ and Zn^2+^ ions and is able to hydrolyse an even wider range of CoA derivatives, including acetoacetyl-CoA [31].

#### The Lower Part of the Paa Pathway

The lower part of the phenylacetate degradation pathway is similar to the fatty acid β-oxidation pathway with the rearrangement of a double bond from Δ^3^ to Δ^2^ position, *i.e.*, Δ^3^-Δ^2^-enoyl-CoA isomerization, hydratation of the double bond, *i.e.*, Δ^2^-enoyl-CoA hydratation, oxidation of the hydroxyl-group to the keto-group and cleavage of the acetyl-CoA.

The product of ring opening by the isomerase PaaG and the hydrolase PaaZ, 3-oxo-5,6-dehydrosuberyl-CoA (compound VI), is shortened by thiolase PaaJ by two carbon atoms to 2,3-dehydroadipyl-CoA (compound VII). The Δ^3^-double bond is rearranged to the Δ^2^-position by PaaG (compound VIII) and hydrated to 3-hydroxyadipyl-CoA (compound IX) by PaaF. Next, dehydrogenase PaaH oxidizes the hydroxyl-group to yield 3-oxoadipyl-CoA (compound X), which is finally cleaved by PaaJ to common metabolites acetyl-CoA and succinyl-CoA [9].

### 3.5. Protein-protein Interactions Among Enzymes of the Lower Part of the Paa Pathway

As enzymes of the fatty acid degradation pathway assemble in multiprotein complexes [32] and the reactions in the lower part of the Paa pathway are similar to fatty acid degradation, we suggested that the enzymes of the lower part may also associate into multiprotein complexes. Methods similar to those described above were used to identify stable complexes among enzymes PaaA, PaaB, PaaC, PaaF, PaaG, PaaH, PaaJ, and PaaZ. In numerous co-expression experiments of up to seven Paa enzymes in a single cell, only one stable complex was identified, that between PaaF and PaaG [33].

### 3.6. Structure of the PaaFG Complex

**Figure 3:**
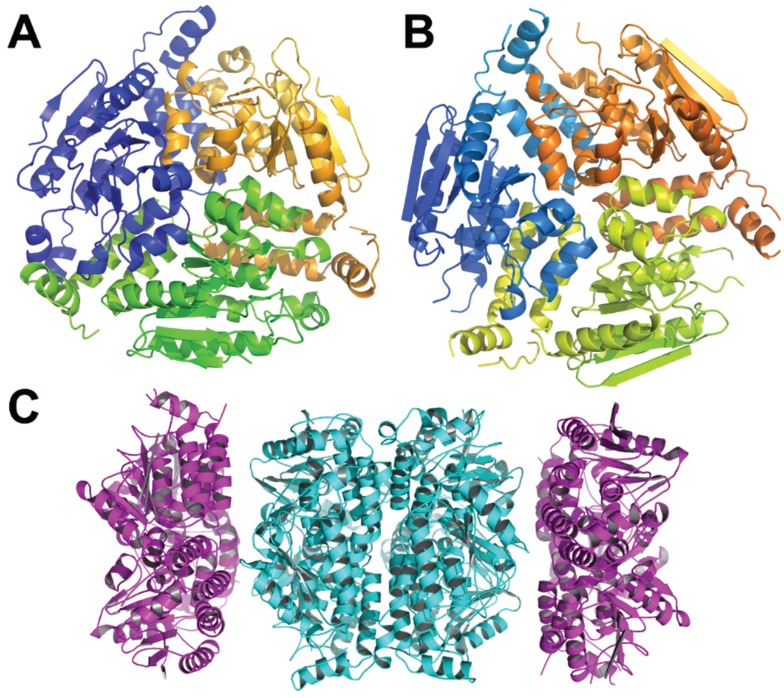
The *E. coli* PaaF-PaaG complex forms a four-layered hetero-dodecamer. (**A**) The trimer of PaaF; (**B**) the trimer of PaaG; (**C**) the arrangement of trimeric rings within the dodecamer. The outside rings (magenta) are PaaG trimers, the inner rings (cyan) are PaaF trimers.

The crystal structure of the PaaF-PaaG complex was determined at 2.5 Å resolution [33]. Both proteins possess a crotonase fold and, as is common for proteins with this fold, they assemble into homotrimeric discs (Figure 3A,B). The complex is composed of a stack of four such discs, two PaaF discs in the center sandwiched between PaaG discs on each end (Figure 3C). In the stack the PaaF disc is positioned between another PaaF disc and a PaaG disc. The latter interacts only with PaaF through one of its flat surfaces, while the other surface is exposed to the solvent. This arrangement is also maintained in solutions, as visualized by negative stain electron microscopy [33].

PaaF and PaaG, like other members of the crotonase superfamily, are folded with repeating ββα-units, which form two almost perpendicular β-sheets, surrounded by α-helices [34]. Since the active sites of PaaF and PaaG are facing the solvent and are located far from each other, the tunnelling hypothesis is unlikely. However, assembling 12 active sites in close proximity within the complex likely speeds up two consecutive reactions along the pathway due to increased local concentration of active sites.

### 3.7. Other Enzymes Involved in the Lower Part of the Paa Pathway

**Figure 4:**
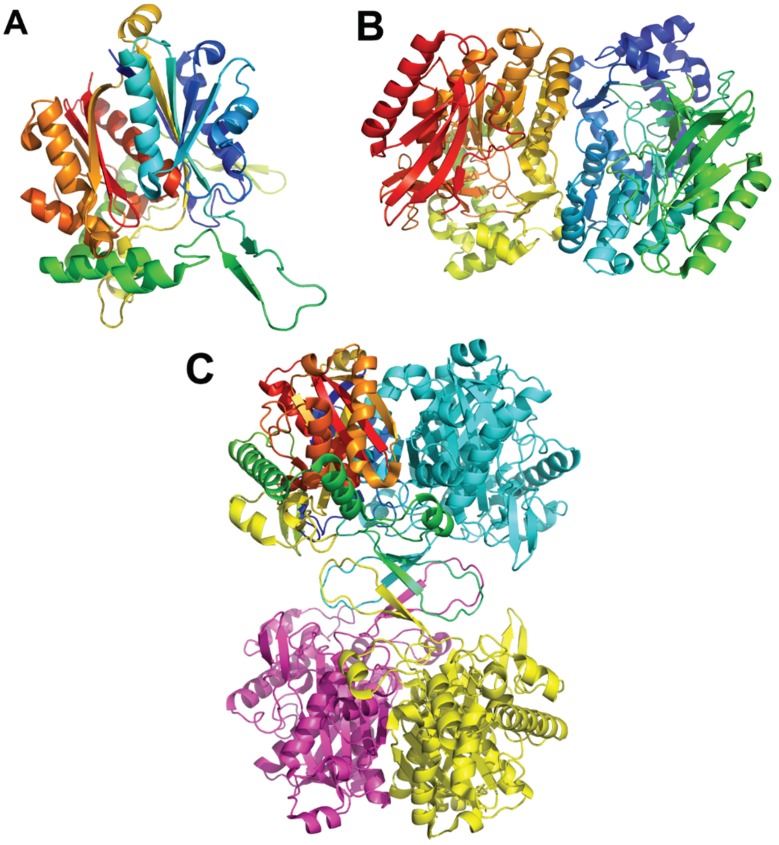
The *Thermus thermophilus* HB8 PaaJ. The biological unit is a dimer of dimers. (**A**) The cartoon representation of the PaaJ monomer painted in rainbow color from blue at the N-terminus to red at the C-terminus. The structure is composed of five layers α/β/α/β/α. (**B**) The cartoon representation of a tightly bound dimer. The second monomer is in cyan. (**C**) The tetramer, third monomer is in magenta and fourth in yellow.

The structures of β-ketoadipyl-CoA thiolase PaaJ from several bacteria have been determined (*Thermus thermophilus*, 1ULQ, 57% identity to *E. coli* PaaJ; *Clostridium acetobutylicum* EA 2018, PDB code 4N44, 45% identity; *Clostridium difficile*, PDB code 4E1L, 44% identity). The monomer has an α/β structure with the central layer of two α-helices sandwiched between four- and five-stranded β-sheets followed by α-helical layers (Figure 4). The monomers assemble into tetramers with two lobes and a central β-barrel composed of elements from four monomers holding them together.

**Figure 5:**
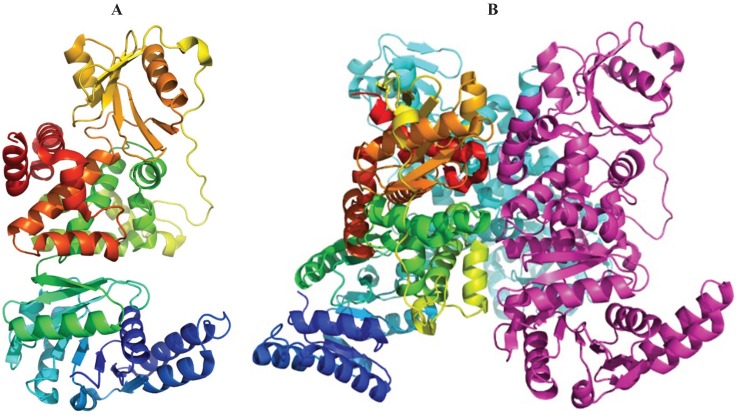
The structure of *E. coli* PaaH. (**A**) The cartoon representation of the monomer painted rainbow colors. The dimerization domain is made of two segments, the one following the N-terminal domain (in green) and the C-terminus (in red); (**B**) the structure of a trimer. The inter-monomer contacts involve mainly the oligomerization domain.

The structure of the 3-hydroxyadipyl-CoA dehydrogenase PaaH from *E. coli* has been recently determined (PDB code 3MOG). This ~500-amino-acid enzyme contains three domains: the N-terminal Rossmann-fold domain, the “dimerization” domain comprising residues 188–280 and 388–475, and a reduced Rossmann-fold domain (residues 305–383) (Figure 5).

#### Other Proteins of the Paa Operon

PaaD is a small protein of around 170 residues, which was shown to be indispensable *in vivo* for the function of PaaABCE monooxygenase complex [8], although *in vitro* its presence was not required for catalysis [9,16]. It is predicted to contain a domain of unknown function DUF59 [35] and shows similarity to SufT, a protein involved in iron-sulfur cluster assembly. The similarity to SufT might indicate that PaaD is involved in the incorporation of iron ions in PaaE or PaaA.

PaaX is a transcriptional regulator to all three open-reading frames of the Paa operon. The binding to DNA is inhibited by phenylacetyl-CoA but not by phenylacetate alone [31,36]. It is also hypothesized that the intermediary CoA derivatives of the Paa catabolic pathway increase transcription inhibition by PaaX, while the inhibition is released by PaaY, which hydrolyses 2-hydroxycyclohepta-1,4,6-triene-1-formyl-CoA and PA-CoA [31].

PaaX has a distant homolog of known structure, that from *Mycobacterium tuberculosis* with 23% sequence identity (PDB code 3KFW), and which structure indicates the presence of the helix-turn-helix DNA-binding motif. PaaX belongs to the GntR family of regulators. Although crystallization of PaaX from *E. coli* was reported [37], no structural data are yet available [31,36].

## 4. Similarities with Other Metabolic Pathways

Steps in the upper part of the Paa pathway are very similar to the degradation of benzoic acid in the Box pathway, where a ligase first attaches the aromatic compound to CoA, the ring is then oxidized by the BoxAB complex to yield a 2,3-epoxybenzoyl-CoA, the ring is then opened by BoxC through a hypothesized formation on an oxepin and the terminal aldehyde group is oxidized by BoxD to yield 3,4-dehydroadipyl-CoA [10].

The action of BoxA and BoxB enzymes, which perform monooxygenation of the benzoyl-CoA, is similar to that of the PaaABCE complex. BoxB is a structural homolog of PaaA, but unlike other monooxygenases that are heteromeric, BoxB does not require a structural subunit and is monomeric [38]. While the di-iron center was empty in the high resolution structure of PaaA, the iron ions were observed in the structure of BoxB together with the substrate benzoic moiety. Both PaaA and BoxB are very specific toward their corresponding substrates and show no cross-reactivity for benzoyl-CoA and phenylacetyl-CoA, correspondingly. Indeed, PaaABCE was inhibited by the addition of benzoyl-CoA [14], while BoxB could not catalyze phenylacetyl-CoA monooxygenation [39].

BoxA is a reductase, which contains two [4Fe-4S] clusters and is specific for NADPH and FAD [38], and thus BoxA does not bear direct similarity to PaaE or to typical reductases of monooxygenases. BoxA does not co-purify with BoxB, and its concentration in living cells is much lower than that of BoxB. Epoxidation of the benzoic ring by BoxB requires only minor amounts of BoxA, suggesting a transient interaction between these two enzymes. However, the enzyme that catalyzes ring opening in the next step of the pathway, BoxC, co-purifies with BoxB suggesting that they form a complex. Moreover, BoxC is essential for BoxB to overcome auto-inhibition with its reaction product 2,3-epoxybenzoyl-CoA [10]. PaaABCE activity is similarly dependent on the downstream enzyme PaaZ [14].

BoxC (2,3-epoxybenzoyl-CoA dihydrolase, PaaF and PaaG homolog) and BoxD (3,4-dehydroadipyl-CoA semialdehyde dehydrogenase, PaaZ homolog), the latter containing only an aldehyde dehydrogenase domain, are involved in epoxide rearrangement and ring opening and oxidation. BoxC contains two homologous crotonase fold domains, interacting with each other in a symmetrical manner, although only the N-terminal domain contains an active center [40]. Its active site, hydrophobic in nature, contains two Glu residues positioned in the same locations as in PaaF. This is in line with the proposed BoxC function for conducting two consecutive hydrolysis steps and a variety of reactions, catalyzed by the crotonase protein family [10]. The structure of BoxD [41] revealed a tightly bound homodimer with each monomer made of three domains: Rossmann-fold cofactor binding domain, the catalytic domain and an oligomerization domain. Both PaaZ and BoxD are NADP^+^-specific enzymes.

The lower part of the Paa pathway is very similar to the fatty acid β-oxidation pathway. In the case of a fatty acid with a double bond, the enoyl-CoA is first isomerized by Δ^3^-Δ^2^-enoyl-CoA isomerase (ECI) and Δ^3,5^-dienoyl-CoA isomerase (DCI), which rearrange the position of one or two conjugated double bonds, correspondingly [42,43,44,45]. The other steps, which involve 2-enoyl-CoA hydratase (ECH), 3-hydroxyacyl-CoA dehydrogenase (HACD) and 3-ketoacyl-CoA thiolase (KACT) activities, may be performed by separate enzymes, multifunctional enzymes or enzyme complexes [32,46,47]. The β-oxidation in bacteria is performed by the αββα heterotetrameric complex (called FOM), where the α-subunit contains domains with 2-enoyl-CoA hydratase (ECH) and 3-hydroxyacyl-CoA dehydrogenase activities (HACD) activities, while the β-subunit is a 3-ketoacyl-CoA thiolase (KACT) [48].

The isomerase PaaG is similar to ECI. The structure of PaaG shows that it possesses only one catalytic glutamate, which is in line with the structure of an active center of isomerases. The shape and properties of the substrate-binding pocket reveal how the enzyme accommodates two structurally diverse substrates to perform two different chemical reactions, the unusual rearrangement of the epoxide to an oxepin, and the rearrangement of the double bond from the Δ^3^ to the Δ^2^ position.

The reaction catalyzed by PaaF, hydration of 2,3-dehydroadipyl-CoA, is similar to that performed by ECH and the comparison of their structures shows that the positioning of the two catalytic glutamates is the same in both enzymes. The shape and charge distribution in PaaF show adaptation for binding of the substrate with a negatively-charged carboxyl group as opposed to a fully hydrophobic alkyl moiety for ECH.

Although the architecture of the PaaFG complex bears no similarity to structures of the fatty acid β-oxidation complex FOM [48] and multifunctional enzyme MFE-1 [32], it proves that monofunctional enzymes can also assemble into highly-oligomeric structures with potential functional benefits.

PaaH appears to be a fusion of two hydroxyacyl-CoA dehydrogenase genes and its structure mimics a dimer of the human heart short chain L-3-hydroxyacyl-CoA dehydrogenase [49]. The two fully functional Rossmann-fold domains are connected together *via* a dimerization domain. However, in the structure of PaaH, only the N-terminal Rossmann-fold domain appears to be fully-functional, while the second Rossmann-fold domain (residues 305–383) is missing the catalytic residues and an NAD(H) binding cleft. By analogy with the human enzyme, the active site is likely to be placed in the cavity between the N-terminal Rossmann-fold domain and a “dimerization” domain. The catalytically important residues His142 and Glu154 are conserved.

Finally, the structure of PaaJ from *Thermus Thermophilus* (PDB code 1ULQ) is very similar to human mitochondrial acetoacetyl-CoA thiolase (2IB7) ([50] with RMSD of 1.0 Å for common 350 C_α_ atoms of the two monomers.

## 5. Conclusions

The phenylacetate (PA) utilization pathway represents the central route for degradation of a variety of organic compounds, whose degradation reactions converge at this pathway. The pathway combines the features of aerobic and anaerobic oxidative metabolic pathways using oxygen to open the aromatic ring and coenzyme A derivatization of phenylacetate. During the last few years the pathway have been characterized and the reactions catalyzed by the Paa enzymes as well as their substrates and products were identified. For many Paa enzymes the structures are known while for the others the structural information can be inferred from the structures of their homologs. Only two protein-protein complexes have been identified among Paa enzymes, that of PaaABCD and PaaFG. The first complex serves an indispensable monooxygenase which inserts one oxygen atom into an aromatic ring to form an epoxide. The same complex has an ability to deoxygenate the epoxide in order to control its intracellular concentration. PaaFG, although being dissimilar to protein complexes and multifunctional enzymes known for a fatty acid β-oxidation pathway, shows a unique assembly of the two enzymes of the crotonase fold with a potential benefit for the speed of the catabolic conversion.
